# Metabolic Marker GLUT1 in Salivary Gland Cancers: Quantification and Effect-Size Estimation

**DOI:** 10.3390/biomedicines14061300

**Published:** 2026-06-08

**Authors:** Wojciech Domka, Maciej Misiołek, Agnieszka Przygórzewska, Tomasz Kubrak, Angelika Myśliwiec, Dorota Bartusik-Aebisher, David Aebisher

**Affiliations:** 1 Department of Otolaryngology, Faculty of Medicine, Collegium Medicum, University of Rzeszów, 35-959 Rzeszów, Poland; 2 Department of Otorhinolaryngology and Oncological Laryngology in Zabrze, Medical University of Silesia, 40-055 Katowice, Poland; maciej.misiolek@sum.edu.pl; 3 Doctoral School, University of Rzeszów, 35-959 Rzeszów, Poland; ap117623@stud.ur.edu.pl; 4 Department of Biochemistry and General Chemistry, Faculty of Medicine, Collegium Medicum, University of Rzeszów, 35-959 Rzeszów, Poland; tkubrak@ur.edu.pl (T.K.); amysliwiec@ur.edu.pl (A.M.); dbartusikaebisher@ur.edu.pl (D.B.-A.); 5 Department of Photomedicine and Physical Chemistry, Faculty of Medicine, University of Rzeszów, 35-959 Rzeszów, Poland

**Keywords:** salivary glands, salivary gland tumors, glucose transporter type 1

## Abstract

**Background**: Glucose transporter 1 (GLUT1) is frequently upregulated in solid tumors and may reflect metabolic adaptation of malignant tissues. However, evidence regarding GLUT1 protein levels in salivary gland tumors remains limited. **Methods**: In this pilot study, GLUT1 protein concentrations were quantified in tissue homogenate supernatants from salivary gland tumors (n = 9) and non-malignant salivary gland tissue obtained from surgical margins (controls; n = 4) using a commercial ELISA kit (BlueGene Biotech; E01G0020) according to the manufacturer’s instructions. Supernatants were stored at −80 °C until analysis. Group comparisons were performed using a non-parametric Mann–Whitney U test. **Results**: GLUT1 levels showed substantial inter-individual variability. The tumor group exhibited higher values than controls [median (IQR): 15.53 (12.44–26.38) vs. 10.14 (7.40–13.26); mean ± SD: 19.26 ± 11.49 vs. 10.33 ± 4.39 (ng/mL)], although the between-group difference did not reach statistical significance (Mann–Whitney U = 27, two-sided *p* = 0.199). **Conclusions**: These preliminary data suggest heterogeneity of GLUT1 levels in salivary gland tumor tissue homogenates and numerically higher concentrations compared with non-malignant margin tissue. These findings should be interpreted as preliminary and hypothesis-generating. Larger, clinically annotated cohorts with orthogonal validation are required before any diagnostic, prognostic, or clinical relevance of GLUT1 can be considered.

## 1. Introduction

Salivary gland malignancies constitute a rare and clinically challenging group of head and neck cancers, accounting for less than 5% of all head and neck cancers. Current reviews indicate that their treatment remains challenging due to the significant variability in clinical course, frequent diagnostic challenges, and limited data regarding optimal systemic treatment in advanced disease. GLUT1 expression has been demonstrated in salivary gland malignancies and has been associated with poor prognosis in tissue studies [1]. Although population-based data indicate a low incidence of these tumors, global data highlight the significant absolute epidemiological burden and suggest the need for improved stratification strategies based on tumor biology; the International Agency for Research on Cancer Global Cancer Observatory [2] reported 53,583 new cases of salivary gland cancer worldwide, corresponding to an incidence of approximately 0.59 per 100,000 persons per year. Apart from their rarity, salivary gland tumors are characterized by significant morphological and biological heterogeneity, encompassing a wide spectrum of histological entities with different molecular mechanisms and diverse progression, which further complicates both diagnosis and prognosis [3,4,5].

In recent years, the molecular characterization of salivary gland malignancies has become particularly important, as classical histopathological approaches prove insufficient to fully predict the clinical course of the disease. Individual histological subtypes exhibit specific genetic abnormalities that have both diagnostic and potential therapeutic implications. For example, secretory salivary gland carcinoma is characterized by the presence of an ETV6-NTRK3 gene fusion, while ductal salivary gland carcinoma often demonstrates androgen receptor overexpression and HER2/ERBB2 amplification, opening the possibility of targeted therapies. Weaver AN et al. emphasize that the identification of such molecular alterations is gradually changing the treatment algorithm for recurrent and metastatic disease, particularly in patients with limited efficacy of classical chemotherapy. The authors indicate that molecular biomarkers are becoming one of the most important elements of modern risk stratification, and their prognostic significance may be comparable to classical histological features, such as the degree of malignancy or the presence of perineural invasion [6,7].

Despite diagnostic advances, systemic treatment of advanced salivary gland cancers remains an area of limited clinical evidence, primarily due to the rarity of the disease and the lack of large randomized clinical trials. A systematic review by Silva et al. demonstrated that most available systemic treatment regimens primarily lead to disease stabilization, while complete and partial response rates remain low. The best results were observed in molecularly selected subgroups, particularly in patients with androgen receptor expression treated with hormone therapy and in patients with HER2 amplification receiving anti-HER2 therapy. Similar conclusions were presented by Prost et al., demonstrating that the effectiveness of systemic treatment is strongly dependent on the histological type and molecular profile of the tumor, further justifying the need for predictive biomarkers, such as GLUT1, that can identify tumors with a more aggressive metabolism and poorer prognosis [8,9].

Remodeling of cancer metabolism is one of the fundamental hallmarks of cancer and involves increased glucose uptake and a shift in cellular metabolism towards glycolysis even in the presence of oxygen, referred to as the Warburg effect [10,11,12]. To maintain this metabolic demand, cancer cells increase their glucose transport capacity primarily through GLUT transporters belonging to the SLC2 family, enabling transmembrane glucose influx and supporting both bioenergetic needs and the synthesis of anabolic precursors [13,14,15]. Of these transporters, GLUT1 (SLC2A1) remains the best-characterized isoform and is frequently associated with the malignant phenotype in many cancer types [16,17]. In cancer, GLUT1 expression can be induced by both oncogenic signaling and microenvironmental stress, including activation of the PI3K/Akt pathway, transcriptional induction by HIF-1α in hypoxic conditions, and c-Myc-dependent programs [18,19,20,21,22,23,24].

**Figure 1:**
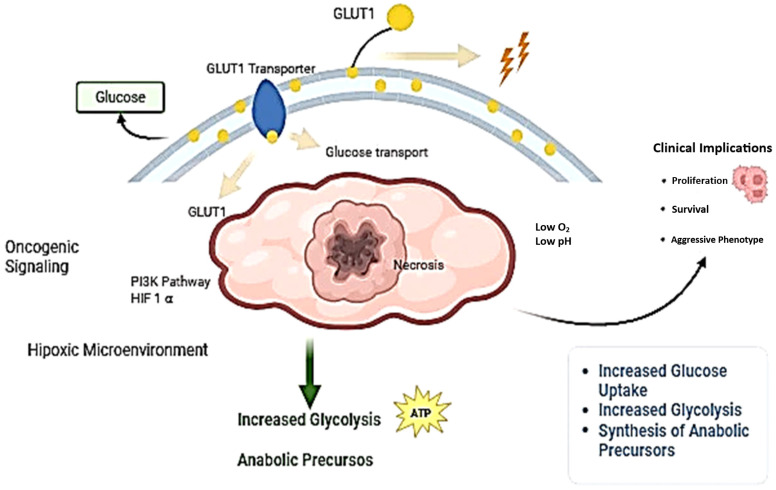
The GLUT1 transporter, located at the plasma membrane, facilitates increased glucose influx into cancer cells. Its expression is induced by oncogenic signals (e.g., the PI3K/Akt pathway, c-Myc) and hypoxic conditions (HIF-1α). Increased glucose transport leads to enhanced glycolysis (Warburg effect), increased ATP production, and synthesis of anabolic precursors, promoting cell proliferation, survival, and an aggressive tumor phenotype.

In addition to its metabolic role, GLUT1 overexpression has been consistently associated with aggressive tumor behavior and poor prognosis in many solid tumors, including head and neck cancers (Figure 1). In head and neck squamous cell carcinomas, elevated GLUT1 expression correlates with higher histological grade, increased proliferative activity, adaptation to hypoxia, and shortened disease-free survival. Ayala FR et al. demonstrated that positive GLUT1 immunoexpression is an independent factor associated with poor survival in oral squamous cell carcinoma [25]. Similar observations were presented by Li S et al., demonstrating the association of GLUT1 expression with survival of patients with head and neck squamous cell carcinoma [26].

An important aspect of GLUT1 biology in salivary gland cancers is its close association with the cancer cell response to hypoxia. In many solid tumors, SLC2A1/GLUT1 expression remains regulated by activation of the HIF-1α axis, which triggers metabolic adaptations to maintain ATP production under oxygen-limited conditions. In head and neck cancers, areas of high GLUT1 expression often coexist with markers of hypoxia and increased resistance to anticancer therapy. This is particularly important in high-grade salivary gland cancers, where irregular vascular architecture and the presence of extensive areas of necrosis may favor local activation of hypoxic programs. Airley RE et al. demonstrated a positive correlation between GLUT1 and HIF-1α expression and the severity of the tumor’s aggressive biological phenotype [27].

Clinically, elevated GLUT1 expression has been associated with unfavorable tumor biology and poorer treatment outcomes in many cancers, and meta-analytic data support its overall prognostic significance [28]. In the case of salivary gland tumors, their histological heterogeneity and variable hypoxic microenvironment may be particularly important, potentially resulting in significant interpatient variability in metabolic pathway activation. Immunohistochemical studies have demonstrated GLUT1 expression in malignant salivary gland tumors and suggest a possible association with clinicopathological aggressiveness and survival [1]. Comparative analyses of different types of salivary gland tumors also indicate differential patterns of GLUT1 immunoexpression and their relationship with angiogenesis [29], and studies examining markers associated with hypoxia and lipid metabolism support the hypothesis that GLUT1 may be part of broader metabolic adaptation programs in these tumors [30]. Nagao T et al. emphasize the usefulness of immunohistochemical markers in differentiating the biology of salivary gland tumors [31].

Despite these observations, quantitative data on GLUT1 protein levels in salivary gland tumor tissue, particularly in supernatants derived from tissue homogenates, remain limited. Analysis of tissue homogenates captures a complex signal originating not only from tumor cells but also from stromal, vascular, and immune components, reflecting the global metabolic adaptation of the tumor microenvironment, although without the ability to localize the signal spatially. Therefore, pilot datasets may be valuable in assessing feasibility, estimating effect sizes, and planning further validation studies integrating orthogonal methods such as immunohistochemistry.

Quantitative protein analysis in tumor tissue homogenates is an important complement to classical immunohistochemical analyses, as it allows for the assessment of the full spectrum of proteins present in both tumor cells and components of the tumor microenvironment, including stromal and vascular components, as well as infiltrating leukocytes. This approach is sometimes used in proteomic studies, where homogenized tissue samples are analyzed using mass spectrometry to identify differences in protein levels between healthy and neoplastic tissues. An example of this approach is the analysis of peptidomes extracted from healthy and neoplastic parotid gland tissues, which revealed the presence of specific peptides present only in tumor materials, suggesting the possibility of identifying candidate cancer biomarkers using quantitative methods in tissue homogenates [32].

Such work highlights the potential of proteomic analyses to uncover unique protein profiles associated with cancer and differences between healthy and tumor tissue, which may also include metabolic markers such as GLUT1. Various cancers tend to overexpress GLUT1 at both the transcriptional and protein levels, as supported by extensive immunohistochemical reviews indicating that GLUT1 is more commonly overexpressed in cancer tissues than in normal tissues; meta-analyses across cancer types show a significant increase in the frequency of GLUT1 expression in cancer tissues compared with normal tissues, particularly in head and neck cancers, suggesting its potential as a prognostic or diagnostic biomarker [33].

Therefore, the aim of this pilot study was to assess differences in GLUT1 levels in supernatants of salivary gland tumor tissue homogenates compared to benign salivary gland tissue collected from surgical margins.

## 2. Materials and Methods

### 2.1. Study Setting and Tissue Samples

This study was conducted at the Department of Otolaryngology, Pediatric Otolaryngology and Laryngological Oncology, University Clinical Hospital No. 1 in Rzeszów (Poland). Tissue samples were obtained during surgical resection. The tumor group comprised salivary gland malignant tumor tissues (n = 9). The tumor cohort included histologically confirmed malignant salivary gland neoplasms representing multiple histological subtypes. Detailed clinicopathological characteristics of the included patients, including histological subtype, tumor grade, anatomical location, and available staging parameters, are presented in Table 1. Control samples consisted of non-malignant salivary gland tissue obtained from surgical margins (n = 4), verified as free of malignant infiltration on routine histopathological assessment. Due to the exploratory pilot nature of the study and the limited cohort size, the study was not designed or statistically powered for subtype-specific analyses.

The study was conducted in accordance with the Declaration of Helsinki and approved by the Bioethics Committee of the District Medical Chamber in Rzeszów of 8 August 2019, approval number: 93/B/2019 with the extension of ethical approval validity until 31 December 2024.

### 2.2. Sample Processing and Preparation of Tissue Homogenate Supernatants

Fresh tissue fragments were snap-frozen in liquid nitrogen immediately after excision and stored at −80 °C until analysis. Prior to homogenization, samples were thawed on ice, briefly rinsed with ice-cold phosphate-buffered saline (PBS, pH 7.2–7.4) to remove residual blood, blotted dry, and weighed (typically 300–500 mg). Tissues were minced and homogenized on ice in ice-cold PBS using a consistent tissue-to-buffer ratio across all samples (1:10 *w*/*v*). Homogenates were subsequently clarified by centrifugation (at 10,000× *g*, 10 min, 4 °C; according to a constant protocol applied to all samples), and the supernatants were collected for GLUT1 quantification.

Because GLUT1 is an integral transmembrane protein, the measured ELISA signal should be interpreted as the fraction of GLUT1 recovered in the clarified tissue homogenate supernatant after mechanical tissue disruption and centrifugation, rather than as a direct measurement of total membrane-bound GLUT1 expression in intact tissue. Tissue homogenization was performed under identical conditions for all samples to disrupt tissue architecture and release cellular and membrane-associated protein fractions into the homogenate. After centrifugation, the collected supernatant represented a soluble/particulate protein-containing fraction suitable for ELISA-based quantification according to the manufacturer’s protocol. Therefore, GLUT1 concentrations reported in this study reflect assay-detectable GLUT1 in tissue homogenate supernatants and should be interpreted as a quantitative tissue-level signal rather than spatially resolved membrane expression.

### 2.3. GLUT1 Quantification by ELISA

GLUT1 concentrations in tissue homogenate supernatants were measured using a commercial enzyme-linked immunosorbent assay (ELISA) kit (BlueGene Biotech, Shanghai, China; Cat. No. E01G0020), following the manufacturer’s instructions. Prior to use, reagents and samples were equilibrated to room temperature (18–25 °C). Briefly, 100 µL of standards or samples were added to wells (blank wells received PBS), followed by addition of the provided conjugate and incubation at 37 °C. After washing (five cycles), substrate solutions were added and incubated for 15–20 min at 37 °C, then the reaction was stopped and absorbance was read at 450 nm using a microplate reader (Tecan Infinite 200 PRO). A standard curve was generated using the kit standards (0, 1.25, 2.5, 5, 10, and 25 ng/mL). All measurements were performed in triplicate, and GLUT1 concentrations were reported as ng/mL of homogenate supernatant.

The ELISA kit used in this study was applied according to the manufacturer’s instructions for human GLUT1 quantification. No independent analytical validation of the assay for salivary gland tissue homogenate supernatants, such as spike-and-recovery, dilution linearity, or comparison with immunohistochemistry/Western blotting, was performed in the present pilot study. This methodological limitation has been explicitly acknowledged in the Discussion.

### 2.4. Statistical Analysis

Given the small sample size, between-group comparisons were performed using a non-parametric Mann–Whitney U test (two-sided). Data are presented as individual values with median and interquartile range (IQR); mean ± standard deviation (SD) is additionally provided for descriptive purposes. Statistical significance was set at α = 0.05.

Because of the pilot nature of this study and the very small sample size, statistical testing was interpreted descriptively and exploratorily. Emphasis was placed on individual values, group distributions, overlap between groups, and exploratory effect-size estimation rather than formal hypothesis confirmation.

## 3. Results

### GLUT1 Levels in Salivary Gland Tumors Show Numerically Higher Values than in Non-Malignant Margin Tissue

**Figure 2:**
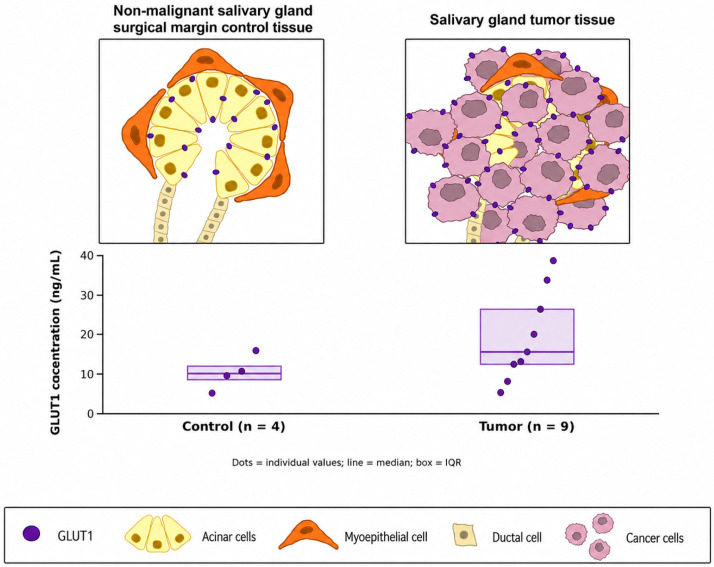
Individual GLUT1 concentrations in tissue homogenate supernatants from non-malignant salivary gland surgical margin controls and salivary gland tumors. Individual values are shown for non-malignant surgical margin control tissue (n = 4) and salivary gland tumor tissue (n = 9). Horizontal lines indicate median values, and boxes indicate interquartile ranges. The tumor group showed numerically higher GLUT1 concentrations than controls, although substantial inter-individual variability and partial overlap between groups were observed. The schematic illustrates the tissue context and predominant cellular compartments; GLUT1 is indicated by purple markers.

GLUT1 concentrations measured in tissue homogenate supernatants showed substantial inter-individual variability in both groups (Table 1; Figure 2). In tumor samples (n = 9), GLUT1 values ranged from 5.32 to 38.75 ng/mL, whereas in control non-malignant salivary gland tissue obtained from surgical margins (n = 4), values ranged from 5.18 to 15.89 ng/mL. Although the distributions partially overlapped, the tumor group exhibited a higher central tendency compared with controls (tumor: mean ± SD 19.26 ± 11.49 ng/mL, median (IQR) 15.53 (12.44–26.38) ng/mL; control: mean ± SD 10.33 ± 4.39 ng/mL, median (IQR) 10.14 (7.40–13.26) ng/mL).

The between-group difference did not reach statistical significance in this small pilot cohort (Mann–Whitney U = 27, two-sided *p* = 0.199). Therefore, the statistical analysis should be interpreted as descriptive and exploratory, with the Hodges–Lehmann median difference serving as an estimate of effect magnitude rather than confirmatory evidence. As an exploratory estimate of effect magnitude, the median difference (Hodges–Lehmann) between tumor and control groups was +6.22 ng/mL (bootstrap 95% CI −0.43 to 20.81), indicating a numerically higher tumor-associated signal but considerable uncertainty due to limited sample size. The observed effect estimate was imprecise, and because the bootstrap confidence interval crossed zero, it should be interpreted cautiously as a hypothesis-generating signal rather than evidence of a confirmed biomarker effect.

Individual GLUT1 concentrations for all samples are provided in Table 1.

## 4. Discussion

In this pilot study, we quantified GLUT1 protein concentrations in tissue homogenate supernatants obtained from salivary gland malignant tumors and non-malignant surgical margin tissue. We observed a numerically higher central tendency of GLUT1 in tumor samples compared with controls, accompanied by substantial inter-individual variability and partial overlap of values between groups. The between-group difference did not reach statistical significance (Mann–Whitney *p* = 0.199), which is not unexpected given the small cohort size and the known biological heterogeneity of salivary gland malignancies [3,4,5]. Collectively, these findings should be interpreted as hypothesis-generating and primarily informative for feasibility and effect-size estimation rather than biomarker validation. These findings provide preliminary feasibility data and suggest that GLUT1 quantification in tissue homogenate supernatants may be further explored in larger, clinically annotated cohorts. The present results should not be interpreted as evidence of diagnostic, prognostic, or clinical utility of GLUT1 in salivary gland tumors.

Our results are directionally consistent with the broader concept of metabolic reprogramming in cancer, where increased glucose uptake and glycolytic reliance provide proliferative and survival advantages (Warburg effect) [10,11]. GLUT1 is a central mediator of glucose influx and is among the best-characterized facilitative glucose transporters, with a well-defined structure and broad physiological distribution [16,17,34,35]. In malignancy, GLUT1 upregulation has been repeatedly linked to aggressive phenotypes and worse outcomes across multiple tumor types [36,37,38,39,40,41,42,43,44,45], with meta-analytic evidence supporting its overall adverse prognostic relevance [28]. Within head and neck oncology, GLUT1 and HK-II have been discussed as contributors to tumor biological behavior and potential biomarkers of aggressiveness [46]. In this context, salivary gland cancers remain an area where clinically useful biomarkers are needed to aid diagnosis, prognostication, and treatment personalization [3,4].

Importantly, salivary gland tumors represent a particularly heterogeneous set of entities, and GLUT1-associated signals may differ by histologic subtype, grade, and microenvironmental stress. Salivary gland-specific studies based on immunohistochemistry provide relevant context. Mori et al. reported that GLUT1 expression in salivary gland tumors was associated with poor prognosis, supporting the notion that higher GLUT1 may reflect aggressive tumor biology [1]. In mucoepidermoid carcinoma, GLUT1 immunoexpression has been shown to correlate with grade of malignancy, further suggesting subtype- and grade-dependent variability [47]. Likewise, de Souza et al. demonstrated differential GLUT1 immunoexpression across pleomorphic adenoma, adenoid cystic carcinoma, and mucoepidermoid carcinoma and additionally linked GLUT1 patterns with an angiogenic index, consistent with heterogeneity in metabolic and vascular adaptations among salivary gland tumors [29]. More recently, Scarini et al. reported coordinated gene and protein-level expression of HIF-1α and GLUT1 (among other metabolic markers) in the development of carcinoma ex pleomorphic adenoma, supporting the integration of GLUT1 into broader hypoxia- and lipid/metabolic programs during salivary gland tumor progression [30]. Taken together, these studies reinforce two key interpretations that match our pilot data: (i) GLUT1 involvement in salivary gland tumor biology is plausible and supported by tissue-based evidence, and (ii) substantial inter-individual dispersion is expected due to histologic and microenvironmental diversity [1,29,30,47].

Mechanistically, tumor-associated GLUT1 upregulation is biologically plausible, as GLUT1 expression may be influenced by oncogenic signaling pathways such as PI3K/Akt and by hypoxia-related metabolic adaptation mediated through HIF-dependent mechanisms [18,19,20,21,22,23,24]. However, in salivary gland malignancies, the contribution of these mechanisms likely varies depending on histological subtype and microenvironmental context [5,29,30].

GLUT1 should be interpreted as one component of broader tumor metabolic adaptation rather than an isolated marker, but the present dataset is insufficient to draw mechanistic conclusions [10,11,12,18,19,20,21,22,23,24].

A key methodological consideration for interpreting our results is that we measured GLUT1 in tissue homogenate supernatants rather than using spatially resolved approaches such as immunohistochemistry. Homogenate-based measurements capture a composite signal arising from malignant epithelial cells and non-malignant components (fibroblasts, endothelial cells, immune infiltrates) that may contribute to the measured protein pool. Consequently, variability in tumor cellularity, stromal fraction, and vascular density can influence measured concentrations and may partially explain the overlap between tumor and control groups. This is particularly relevant in salivary gland tumors, where the microenvironment and vascular biology have been emphasized as clinically meaningful, including angiogenesis-related features that may intersect with metabolic remodeling [29,48]. From this perspective, homogenate-based GLUT1 quantification may reflect a global tissue microenvironmental phenotype but cannot localize GLUT1 expression to specific compartments or distinguish whether observed differences are driven by tumor epithelium versus surrounding tissue.

Another important limitation concerns the use of non-malignant surgical margin tissue as the control group. Although these margins were histopathologically free of malignant infiltration, they cannot be considered fully equivalent to truly normal salivary gland tissue. Surgical margin samples may still be affected by tumor-adjacent field effects, local inflammation, reactive stromal changes, ischemic alterations related to the surgical procedure, or other microenvironmental modifications within the tumor-bearing gland. These factors could potentially influence GLUT1 levels and increase variability in the control group. Therefore, the comparison between tumor tissue and surgical margin tissue should be interpreted with caution, and future studies should include independent normal salivary gland tissue controls whenever ethically and clinically feasible [49,50].

Our results should also be considered within the context of sample type and control definition. Controls in our study were derived from non-malignant surgical margins. While margins were histopathologically free of malignant infiltration, margin tissue may still differ from truly healthy salivary gland tissue due to local inflammation, reactive changes, fibrosis, or field effects related to the tumor-bearing gland and surgical context. These factors could increase variability in the control group and potentially attenuate apparent differences. In addition, the small control sample size (n = 4) limits precision and increases uncertainty, as reflected by the wide confidence interval of the exploratory effect estimate. As such, our pilot data cannot establish diagnostic cutoffs or clinical discrimination, and the observed numerical difference should be interpreted cautiously.

Despite these limitations, the pilot signal observed here is clinically and biologically plausible, consistent with salivary gland tumor literature linking GLUT1 immunoexpression with aggressiveness and outcomes [1,29,47] and with general cancer metabolism concepts [10,11,15,23,28]. The main contribution of this study is providing preliminary quantitative information on GLUT1 concentrations in salivary gland tumor homogenate supernatants and highlighting substantial inter-individual heterogeneity, which is critical for planning adequately powered validation studies. Future work should expand cohort size and incorporate clinical annotation (histologic subtype, grade, stage, perineural invasion, recurrence/metastasis) to determine whether GLUT1 differs systematically between entities or correlates with adverse features. Orthogonal validation using immunohistochemistry with standardized scoring would clarify spatial localization and enable comparison with the salivary gland-specific literature [1,29,47,51]. Future studies may also benefit from integrating quantitative proteomics approaches and spatial analysis techniques to better characterize the distribution and abundance of GLUT1. Furthermore, incorporating pathway-oriented markers—such as p-AKT in adenoid cystic carcinoma [52] and hypoxia-related markers where appropriate [30,53]—could help disentangle whether GLUT1 reflects predominant oncogenic signaling, microenvironmental stress, or a combination of both. In parallel, normalization strategies (e.g., to total protein content) and standardized tissue-to-buffer ratios may reduce technical variability and improve comparability across samples.

Although GLUT1 is a biologically plausible marker of tumor metabolic adaptation, the present data do not support its diagnostic or prognostic use in salivary gland tumors. The observed numerical differences should be interpreted strictly as exploratory and hypothesis-generating. At this stage, these findings do not establish diagnostic cut-offs, clinical discrimination, prognostic value, or biomarker validity. Therefore, no clinical application of GLUT1 can be recommended based on the current dataset [54].

A major limitation of this study is the very small sample size, particularly the imbalance between tumor samples (n = 9) and controls (n = 4), which substantially limits statistical power. Therefore, the absence of statistical significance should not be interpreted as evidence of no biological difference between groups. Rather, the study is at risk of a type II error, meaning that a potentially relevant difference could remain undetected due to insufficient sample size. This issue is especially important in salivary gland tumors, which are rare and biologically heterogeneous, making small single-center cohorts vulnerable to sampling bias and limited generalizability.

An important limitation of the present study is the marked clinicopathological heterogeneity of the included salivary gland malignancies. These tumors represent biologically distinct entities with potentially different metabolic phenotypes, GLUT1 expression patterns, and clinical behavior. Due to the small pilot cohort, meaningful subgroup analyses according to histological subtype, grade, stage, or anatomical location were not feasible. Therefore, the present findings should be interpreted as an overall exploratory signal rather than evidence applicable uniformly across all salivary gland malignancies [55,56].

Without a clinicopathological correlation, the biological significance of the observed GLUT1 variability remains unclear. Due to the limited sample size, we were unable to reliably assess associations between GLUT1 levels and histological subtype, tumor grade, perineural invasion, recurrence, treatment response, or survival outcomes. Future studies should include larger, clinically annotated cohorts to determine whether GLUT1 variability is associated with clinically relevant pathological features or patient outcomes.

A further limitation is the lack of immunohistochemical validation. Because GLUT1 was quantified in tissue homogenate supernatants, the present approach does not preserve spatial information and cannot determine the cellular or compartmental origin of the measured signal. Therefore, it remains unclear whether the observed GLUT1 concentrations primarily reflect expression in malignant epithelial cells, stromal components, vascular elements, inflammatory infiltrates, or a combination of these compartments. This substantially limits spatial biological interpretation and prevents direct comparison with immunohistochemistry-based studies. Future investigations should incorporate orthogonal validation using immunohistochemistry with standardized scoring to confirm GLUT1 localization and expression patterns. Where feasible, spatially resolved approaches, such as multiplex immunostaining or spatial transcriptomics, could further clarify whether GLUT1 reflects tumor-cell metabolism, stromal remodeling, vascular density, inflammatory infiltration, or broader microenvironmental adaptation [57,58].

A further methodological limitation concerns the biological interpretation of GLUT1 quantification in tissue homogenate supernatants. GLUT1 is an integral transmembrane glucose transporter, and therefore ELISA-based measurement in clarified homogenate supernatants does not directly correspond to spatially preserved membrane expression in intact tumor cells. The measured concentration should instead be interpreted as the assay-detectable fraction of GLUT1 recovered after tissue disruption, homogenization, and centrifugation. This signal may be influenced by the efficiency of membrane disruption, the proportion of tumor cells, stromal and vascular components, and the presence of membrane-derived protein fractions in the homogenate. Moreover, the ELISA was not independently validated in salivary gland tissue homogenates by spike-and-recovery, dilution linearity, Western blotting, or immunohistochemistry. Therefore, the present results should be considered preliminary quantitative homogenate-based data and require orthogonal validation using spatially resolved methods [54,59,60,61].

Another methodological limitation is that GLUT1 concentrations were reported per volume of homogenate supernatant rather than normalized to total protein content. Although all samples were processed using a standardized protocol, including tissue weighing and a constant tissue-to-buffer ratio, this approach does not account for potential differences in total protein yield between homogenates. Such differences may result from variable tumor cellularity, stromal content, vascular components, extracellular matrix composition, or inflammatory infiltration. Therefore, the lack of normalization to total protein content may have increased technical variability and reduced comparability between samples. Protein normalization, for example, by reporting GLUT1 concentrations as ng/mg total protein after BCA or Bradford assay, would likely improve reproducibility and provide a more robust basis for comparisons across heterogeneous tissue homogenates. Future studies should incorporate total protein normalization alongside standardized tissue processing protocols [54].

The exploratory Hodges–Lehmann median difference suggested numerically higher GLUT1 concentrations in tumor tissue; however, the bootstrap confidence interval crossed zero, indicating considerable uncertainty around the estimated effect. Consequently, this estimate should be interpreted only as preliminary and hypothesis-generating, not as evidence supporting GLUT1 as a validated diagnostic or prognostic biomarker. The present data are more appropriately viewed as feasibility information that may help inform sample-size planning for future studies.

Future investigations should include larger and clinically annotated cohorts, ideally collected through multicenter collaboration to overcome the rarity of salivary gland malignancies. Such studies should stratify cases by histological subtype, tumor grade, stage, and clinically relevant outcomes such as recurrence or metastasis. Orthogonal validation using immunohistochemistry would also be necessary to confirm the cellular localization of GLUT1 expression and to determine whether homogenate-based GLUT1 levels reflect tumor epithelial expression, stromal contribution, vascular density, or broader microenvironmental changes. Until such validation is performed, the clinical relevance of GLUT1 in salivary gland tumors remains uncertain.

In conclusion, this pilot analysis suggests that GLUT1 levels in salivary gland tumor tissue homogenate supernatants can be numerically higher than in non-malignant surgical margin tissue, but the difference is not statistically significant in a small cohort and values show notable overlap. These findings provide preliminary feasibility data and may help inform the design of larger, clinically annotated validation studies [1,3,4,29,30,47]. No diagnostic, prognostic, or clinical conclusions can be drawn from the present dataset.

## 5. Conclusions

This pilot study provides preliminary quantitative data on GLUT1 concentrations in salivary gland tumor tissue homogenate supernatants compared with non-malignant surgical margin tissue. Tumor samples showed numerically higher GLUT1 levels with substantial inter-individual variability and overlapping values between groups, and the between-group difference was not statistically significant in this small cohort. Given the limited sample size, use of surgical margins as controls, and the composite nature of homogenate-based measurements without spatial localization, these findings should be considered hypothesis-generating. These findings provide preliminary feasibility data and suggest that GLUT1 quantification in tissue homogenate supernatants may be further explored in larger, clinically annotated cohorts, enabling stratification according to histological subtype, tumor grade, stage, and outcome. No diagnostic, prognostic, or clinical conclusions can be drawn from the present dataset.

## Figures and Tables

**Table 1:** GLUT1 concentrations (ng/mL) in tissue homogenate supernatants from salivary gland tumors and non-malignant surgical margin tissue (controls).

Group	n	Min–Max (ng/mL)	Mean ± SD (ng/mL)	Median (IQR) (ng/mL)
**Salivary gland tumor tissue**	9	5.32–38.75	19.26 ± 11.49	15.53 (12.44–26.38)
**Surgical margin tissue (control)**	4	5.18–15.89	10.33 ± 4.39	10.14 (7.40–13.26)

## Data Availability

The original contributions presented in this study are included in the article/Supplementary Material Tables S1 and S2. Further inquiries can be directed to the corresponding authors.

## Supplementary Materials

The following supporting information can be downloaded at https://www.mdpi.com/article/10.3390/biomedicines14061300/s1. Table S1. Distribution of salivary gland tumor samples according to histopathological subtype. Table S2. Histopathological characteristics of individual salivary gland tumor samples, including grade, stage, and anatomical location.
