# Supplementary material for: Metabolic Marker GLUT1 in Salivary Gland Cancers: Quantification and Effect-Size Estimation
Source: Biomedicines. 2026 Jun 8;14(6):1300. doi: 10.3390/biomedicines14061300 (PMC13297489; doi:10.3390/biomedicines14061300)
Supplement: Supplementary file 1 [file biomedicines-14-01300-s001.zip › biomedicines-4304776-supplementary.pdf]

Supplementary Materials

Table S1. Distribution of salivary gland tumor samples according to histopathological subtype.

Group	n
Mucoepidermoid carcinoma	3
Adenoid cystic carcinoma	1
Acinic cell carcinoma	2
Salivary duct carcinoma	3

Table S2. Histopathological characteristics of individual salivary gland tumor samples, including grade, stage, and anatomical location.

Sample	Histology	Grade	Stage	Location
1	Mucoepidermoid carcinoma	High	III	Parotid
2	Adenoid cystic carcinoma	II	II	Submandibular
3	Acinic cell carcinoma	High-grade transformation	III	Parotid
4	Mucoepidermoid carcinoma	Intermediate	III	Parotid
5	Acinic cell carcinoma	High-grade transformation	II	Parotid
6	Mucoepidermoid carcinoma	High	III	Parotid
7	Salivary duct carcinoma	High	III	Parotid
8	Salivary duct carcinoma	High	III	Parotid
9	Salivary duct carcinoma	High	II	Parotid
